# Full Genome Sequence of a Recombinant H1N2 Avian Influenza Virus Isolated from Wild Waterfowl in the East Dongting Lake Wetland

**DOI:** 10.1128/genomeA.00023-14

**Published:** 2014-02-20

**Authors:** Yun Zhu, Yuesi Huang, Tao Chen, Wenfei Zhu, Changxing Lou, Huahong Wang, Meng Li, Fuqiang Liu, Xingyu Xiang, Linhui Li, Hong Zhang, Yuelong Shu

**Affiliations:** a National Institute for Viral Disease Control and Prevention, China CDC, Key Laboratory for Medical Virology, National Health and Family Planning Commission, Beijing, China; b Hunan Provincial Center for Disease Control and Prevention, Changsha, China; c Yueyang Center for Disease Control and Prevention, Yueyang, China; d Hunan East Dongting Lake National Nature Reserve, Yueyang, China; e Yueyang County Center for Disease Control and Prevention, Yueyang, China

## Abstract

Here, we report the full genome sequence of an H1N2 avian influenza virus (AIV) isolated from wild waterfowl in Dongting Lake. Phylogenetic analysis showed that it was a novel recombinant AIV between domestic ducks and wild waterfowl. Investigation of this virus is helpful for our understanding of the ecology of AIV in this region.

## GENOME ANNOUNCEMENT

Wild waterfowl are recognized as the natural reservoir of influenza A virus, and all of the H1 through H16 and N1 through N9 subtype avian influenza viruses (AIVs) have been identified from them (1, 2). The AIVs circulating in wild birds can occasionally be introduced into domestic poultry and undergo wide reassortment with the AIVs circulating in poultry (3). The H1 subtype influenza viruses have been circulating in avian, swine, and human populations and have caused several pandemics in humans (4). The H1N1 influenza viruses that caused the 1918 Spanish influenza pandemic were thought to have originated from avian influenza viruses (5), so the H1 subtype AIVs circulating in avian populations should not be neglected.

In this study, an H1N2 subtype AIV, named A/wild waterfowl/Dongting/C2383/2012 (H1N2), was isolated from feces of wild waterfowl in the Hunan East Dongting Lake National Nature Reserve in January 2012. To our knowledge, this is the first report of the isolation of an H1N2 subtype AIV in this region. To understand its genetic characterization, we amplified its eight gene segments with the primers designed by our lab and performed full genome sequencing by use of an ABI 3730xl DNA analyzer. The complete viral genome consists of 8 gene segments of negative-sense, single-stranded RNA, encoding PB2, PB1, PA, HA, NP, NA, M, and NS, with corresponding nucleotide lengths of 2,341, 2,341, 2,233, 1,777, 1,565, 1,466, 1,027, and 890 bp. The hemagglutinin (HA) cleavage site possesses only a single basic amino acid, R (PSIQSR↓GLF), indicating low pathogenic effects in poultry (6). The QSG in 226 to 228 (H3 numbering) of HA protein indicated an avian-like receptor binding preference (7). Analysis of the potential glycosylation site motif N-X-S/T revealed six sites, at positions 28, 40, 104, 304, 498, and 557, in HA and eight sites, at positions 61, 69, 70, 86, 146, 200, 234, and 402, in NA. The amino acid residues 627 and 701 of the PB2 protein were E and D, respectively, which indicated that the virus was of avian origin (8, 9).

Phylogenetic analysis revealed that all eight gene segments belonged to the Eurasian gene pool. The HA and NA genes were most closely associated with the viruses isolated from domestic ducks in Guangxi Province, China, and the internal genes showed high nucleotide identity with those of the viruses circulating in wild birds in Asia. Interestingly, the PB2, PB1, and NP genes shared high homology (98% to 99%) with those of A/wild goose/Dongting/C1037/2011(H12N8), which we previously reported in this region (10). These results indicated that the virus underwent wide reassortment with the viruses circulating in domestic poultry and wild birds.

In conclusion, the H1N2 AIV is a novel reassortant virus between domestic poultry and wild waterfowl. Our results are useful for understanding the cross-species of AIV between domestic poultry and wild birds and highlight the importance for pandemic preparedness of AIV surveillance in wild birds and poultry.

### Nucleotide sequence accession numbers.

The genome sequences of A/wild waterfowl/Dongting/C2383/2012 (H1N2) have been deposited in GenBank under accession numbers KF874478 to KF874485.
